# The relationship between mindfulness and empathy with the oxytocinergic system in persons with schizophrenia spectrum disorders – A proof-of-concept randomized controlled trial (OXYGEN)^☆^

**DOI:** 10.1016/j.ijchp.2024.100503

**Published:** 2024-09-10

**Authors:** Kerem Böge, Niklas Bergmann, Marco Zierhut, Inge Hahne, Alice Braun, Julia Kraft, Ingmar Conell, Thi Minh Tam Ta, Neil Thomas, Paul Chadwick, Stephan Ripke, Eric Hahn

**Affiliations:** a Department of Psychiatry and Neuroscience, Campus Benjamin Franklin, Charité – Universitätsmedizin Berlin, Germany; b Department of Psychiatry and Neuroscience, Campus Charité Mitte, Charité – Universitätsmedizin Berlin, Germany; c Centre for Mental Health, Swinburne University of Technology, Melbourne, Australia; d Department of Psychology, University of Bath, Bath, UK; e Stanley Center for Psychiatric Research at Broad Institute of MIT and Harvard, Cambridge, MA, USA; f German Mental Health Center (DZPG), Germany

**Keywords:** Mindfulness, Empathy, Schizophrenia, Psychosis, Randomized controlled trial

## Abstract

**Background:**

The present study explored the feasibility and acceptability as well as the impact of mindfulness-based group therapy (MBGT) on oxytocin levels (OXT) and clinical parameters in outpatients with schizophrenia spectrum disorders (SSD).

**Methods:**

In a randomized-controlled design, outpatients with SSD (*N* = 48) were assigned to either MBGT in addition to German university-level treatment as usual (MBGT+TAU; *n* = 25) or TAU (*n* = 23). At baseline and at four-week post-intervention, clinical parameters and OXT levels were determined.

**Results:**

Results indicate high feasibility and acceptance with a 95.7% adherence- and 94% retention- rate of MBGT in SSD. While no significant changes in empathy were observed, MBGT+TAU demonstrated a significant reduction in positive symptoms (Positive and Negative Syndrom Scale) compared to TAU at post-intervention. OXT levels were significantly increased in MBGT+TAU at post-intervention, suggesting a potential link between mindfulness and the oxytocinergic system in SSD. Additionally, improvements in various clinical parameters were indicated.

**Conclusion:**

The study contributes to the growing evidence supporting feasibility, acceptability, and positive effects of MBGT in outpatients with SSD, emphasizing the need for further research to solidify these findings. Overall, this work sheds first evidence on the intersection of mindfulness, oxytocin, and clinical outcomes in SSD.

## Introduction

In recent years, the need for innovative forms of psychotherapy has prompted the development of novel third-wave CBT-based therapies that promote mindfulness, acceptance of distressing experiences, and self-compassion (Morris, Johns & Oliver, 2013). These mindfulness-based interventions (MBI) have proven their efficacy in treating various mental disorders and have been incorporated into treatment guidelines (National Institute for Health and Care Excellence (NICE), 2014; Schneider, Härter & Schorr, 2017). For schizophrenia spectrum disorders (SSD), current meta-analyses demonstrate the efficacy of MBI on positive- and negative symptoms, affective symptoms, social functioning, and quality of life (Jansen, Gleeson, Bendall, Rice & Alvarez-Jimenez, 2020; Liu, Li & Hsiao, 2021), while there has been no evidence of psychotic symptoms being exacerbated (Böge, Catena, & Hahn, 2022; Böge, Thomas & Jacobsen, 2021). It could be shown that MBI showed robust improvements for negative symptoms, social functioning, and long-term symptom reduction, specifically when delivered in a group format (Jansen et al., 2020). In line with previous research, our working group established strong feasibility and acceptability of mindfulness-based group therapy (MBGT), a novel participatively developed psychological intervention for SSD with a protocol adherence of 95%, a retention rate of 95%, drop-out rates of 5%, and 96% of sessions completed, indicating high treatment fidelity and supporting acceptability and feasibility. Moreover, results showed robust and clinically meaningful improvements in positive- and negative symptoms, depressive symptoms, social functioning, quality of life, and mindfulness (Böge et al., 2021). At the same time, persons with SSD stated increased meta-cognition, empathy, self-efficacy, and self-compassion concerning therapeutic processes at action through qualitative interviews (Böge et al., 2020a, Böge et al., 2021).

Recent research has indicated that MBI have a potential effect on oxytocin (OXT) levels in the blood, a neuropeptide linked to increased empathy in healthy participants (Bellosta-Batalla et al., 2020; Wang et al., 2019). OXT, in general, has previously been associated with pro-social behavior and reduced negative affect in different populations (Peled-Avron, Abu-Akel & Shamay-Tsoory, 2020). In addition, OXT has been found to regulate brain regions involved in the pathophysiology of SSD (Davis, 1980). It has been demonstrated that OXT increases the functional connectivity between the systems for social reward expectation and the network for socio-emotional processes in the brain, which leads to increased social activation and improved social cognition on a behavioral level (Goh, Chen & Lane, 2021; Love, 2014). SSD and, in particular, negative symptoms are associated with deficits in social cognition (Korann et al., 2022), including empathy, which can be seen as particularly important for the subjective quality of life and social functioning of this clinical population (Marder & Galderisi, 2017).

Against this background, the present study investigates whether the findings of Bellosta-Batalla et al. (2020) and Wang et al. (2019) can be replicated in people with SSD. Therefore, the aim of the present proof-of-concept study is, for the first time, to explore the effect of MBGT on basal OXT levels in blood serum and empathy levels in individuals with SSD. Furthermore, in line with previous research, we will examine possible changes in positive- and negative symptoms, depression, anxiety, social functioning, and mindfulness at a within- and between-group level.

## Methods

### Design

This study is a parallel-group, proof-of-concept randomized controlled trial. According to international recommendations and our initial pre-post pilot study with conservative estimates (Böge et al., 2021), *N* = 48 participants were allocated to two trial arms. Following the initial screening for eligibility, participants provided their informed consent and underwent baseline assessments. Recruitment occurred either at the outpatient facility of the Charité – Universititätsmedizin Berlin, Campus Benjamin Franklin, or through responses to flyers distributed in external outpatient facilities, psychiatric and psychotherapeutic practices, assisted living facilities, and psychiatric day hospitals.

**Fig. 1:**
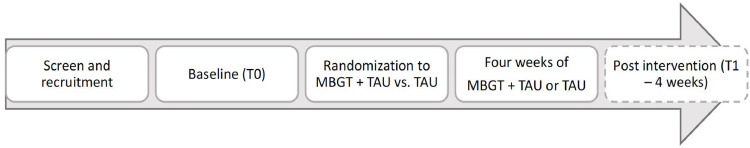
Illustration of the participant flow.

After the baseline assessments, participants were randomly assigned to one of two groups: the experimental group, which received MBGT in addition to their usual treatment (MBGT+TAU; *n* = 25), or the control group, which solely continued with their regular treatment (TAU; *n* = 23). An independent researcher carried out the randomization process using a 1:1 scheme and a fixed block size based on a computer-generated electronic form. Over four weeks, participants attended weekly 60-minute MBGT sessions in addition to their ongoing treatment or consistently received their usual treatment. After the treatment period of four weeks, post-intervention assessments took place. Participants received financial compensation after the completion of the post-intervention assessment. An illustration of the participant flow is displayed in Fig. 1. The study received approval from the ethics committee of the Charité – Universitätsmedizin Berlin (EA4/233/21) and has been registered on clinicaltrials.gov (NCT05491486).

### Inclusion and exclusion criteria

The eligibility criteria for participants were as follows: a) age between 18 and 65 years, b) a confirmed diagnosis of a schizophrenia spectrum disorder (ICD-10: F2X.X) by a qualified psychiatrist, c) adequate proficiency in the German language to participate in the intervention, d) no significant alteration in psychopharmacologic medication within the last six weeks, and e) the ability to provide written informed consent. Exclusion criteria encompassed: a) a score of 7 on any item of the positive scale of the PANSS, indicating severe psychotic symptoms, b) current suicidal tendencies, c) concurrent use of substances other than nicotine, or d) the presence of neurological disorders or brain damage. Current medication, including the dose of antipsychotic drugs measured in haloperidol equivalents, was systematically recorded, aiming for stable dosing during the participation in the study.

### Interventions

*Mindfulness-based Group Therapy.* MBGT was integrated into the experimental condition, where participants underwent a four-week MBGT program in conjunction with their regular treatment at the university hospital outpatient facility. MBGT represents an innovative treatment approach for individuals with SSD, which was specifically crafted through an iterative and participatory research process involving patients with SSD conducted by our research group in recent years (Böge & Hahn, 2021; Böge et al., 2020a, Böge et al., 2021). This program's fundamental modules draw from mindfulness-based cognitive therapy and consider both the original recommendations by Chadwick (2006) for implementing mindfulness in psychosis as well as valuable input from patients. Throughout the intervention, participants delved into various mindfulness facets with a specific focus in each week's session, including breath awareness, sensory perception, detachment, and body awareness. An overview of the content and structure of the MBGT sessions is presented in Table 1. For instance, therapists are advised to avoid cognitive restructuring as outlined in traditional cognitive-behavioral theory. Instead, they should employ the "inquiry principle," which promotes participant sharing experiences to promote perspective-taking and facilitate the intrinsic questioning of maladaptive beliefs (Böge et al., 2021; Heidenreich, Grober & Michalak, 2015). A psychotherapist in training, specializing in cognitive behavioral therapy and possessing over three years of experience of MBI as well as mindfulness practice, led the therapy sessions. The MBGT sessions were closely supervised by an experienced psychotherapist with more than a decade of experience in mindfulness-based therapeutic approaches. During the sessions, meditation intervals were kept short to prevent extended periods of silence, employing basic anchoring techniques and straightforward language by established recommendations (Böge & Hahn, 2021; Chadwick, 2006; Shonin et al., 2014). Unlike standard Mindfulness-Based Stress Reduction (MBSR) and Mindfulness-Based Cognitive Therapy (MBCT) practices, participants were not required to engage in mindfulness exercises between sessions. Nevertheless, they were encouraged to practice mindfulness outside the sessions by distributing handouts summarizing session content and exercises. Participants were also encouraged to set a small personal mindfulness-related goal for each week.

**Table 1:** Mindfulness-based Group Therapy (MBGT) session overview.

Session 1: Mindfulness of breathing	• Introduction and group set-up • Exercise & psychoeducation: Vagal breathing • Psychoeducation: The basics of mindfulness & mindfulness of breathing • Exercise: Using our breath as an anchor • Session wrap-up and weekly goal
Session 2: Mindfulness in nature	• Review of out of session mindfulness goals • Psychoeducation: Biophilia- and attention theory • Exercise: Practicing mindfulness in nature • Psychoeducation: Use of our senses in nature • Exercise: 3–2–1 (“Three things I see, three things I hear…”) • Session wrap-up and weekly goal
Session 3: Mindfulness of Detachment	• Review of out of session mindfulness goals • Psychoeducation: Our recording apparatus • Psychoeducation: The process of letting go • Exercise: Mindfulness of detachment • Exercise: Letting go • Session wrap-up and weekly goal
Session 4: Mindfulness of body awareness	• Review of out of session mindfulness goals • Exercise: Our feet touch the ground • Psychoeducation: Mindfulness of body awareness • Exercise: Body scan • Overall wrap-up and feedback on group

*Treatment as usual.* Most participants (*n* = 37) were enrolled in the outpatient department at Charité – Universitätsmedizin Berlin, Campus Benjamin Franklin. Within this outpatient setting, they received routine care, encompassing monthly consultations with a psychiatrist and pharmacological treatment, sessions with social workers, and individual therapy provided by a psychotherapist or a psychiatric nurse. The remaining participants (*n* = 11) were recruited from other external outpatient facilities, psychiatric and psychotherapeutic practices, assisted living facilities, and psychiatric day clinics in Berlin. Current clinical care was assessed at T_0_ and T_1_ to identify possible differences in treatment. Participation in additional psychotherapy, the type of therapy (individual, group, couple, or family therapy), as well as the frequency (never, once, as needed, weekly, and daily) was assessed. Hence, the received TAU was recorded in detail to be controlled for using chi-square tests and *t*-tests. Furthermore, irrespective of the assigned study condition, all participants received high-quality healthcare services at a renowned university hospital outpatient center, adhering to official national and international treatment guidelines (Gaebel, Hasan & Falkai, 2019; National Institute for Health and Care Excellence (NICE), 2014). This comprehensive care includes access to pharmacological treatment, psychological consultations, and as-needed psychosocial support delivered by trained social workers.

### Assessment

*Oxytocin.* In the MBGT+TAU group, blood samples were collected before and after the first and the fourth MBGT session, summing up to four assessments. In the TAU group, blood samples were taken at two-time points: at baseline (before the first MBGT sessions) and post-intervention (after the fourth MBGT session). For comparability reasons, it was ensured that the time of the day at which the blood samples were taken from participants in the TAU group matched those of the MBGT+TAU group. For plasma Ethylenediaminetetraacetic acid (EDTA) Monovette tubes (Sarstedt, Nümbrecht, Germany) containing aprotinin 400 IU/ml blood were used to avoid hormone degradation. Samples were kept on ice for up to a maximum of 20 min until centrifugation at 1300 g for 10 min at 4 °C. Supernatants were collected and stored immediately at −20 °C for a maximum of six months until OXT levels (pg/ml) were analyzed by a highly sensitive (0.5 pg/ml range) and specific (<0.7% cross-reactivity to a variety of peptides) radioimmunoassay with intra- and inter-assay variabilities of less than 10% (RIAgnosis, Munich, Germany) (Neumann, Maloumby, Beiderbeck, Lukas & Landgraf, 2013). Numerous studies have standardized and validated the utilized assay (Neumann et al., 2013). Prolactin, estrogen, and progesterone levels in venous blood were additionally determined to rule out possible interactions with OXT levels.

Initially, when the study was preregistered on clinicaltrials.gov, an investigation of polygenic risk scores (PRS) for empathy levels was planned. However, the sample size did not allow for such analyses. Further recruitment is currently conducted to cross-sectionally assess whether certain genomes are associated with empathy scores in people with SSD, and outcomes will be published in upcoming articles.

*Measures.* An overview of the study instruments can be seen in Table 2 and the various sociodemographic assessments in Table 3. Gender was assessed providing three choices: male, female, non-binary. Also, prior experience in mindfulness practice has been assessed.

**Table 2:** Study instruments.

	Baseline (T_0_)	Post-intervention (T_1_)
Demographics	x	
Clinical treatment regime	x	x
Blood samples	Before and after MBGT session 1 & 4*	
Empathy Quotient (EQ)	x	x
Interpersonal Reactivity Index (IRI)	x	x
Personal and Social Performance Scale (PSP)	x	x
Positive and Negative Syndrome Scale (PANSS)	x	x
Self-Evaluation of Negative Symptoms (SNS)	x	x
Depression Anxiety Stress Scale (DASS)	x	x
Southampton Mindfulness Questionnaire (SMQ)	x	x
Cognitive Fusion Questionnaire (CFQ)	x	x
Adverse events (AE) and serious adverse events (SAE)		x

Note.

⁎ In the MBGT + TAU group, blood samples were collected both before and after the initial and final MBGT sessions, resulting in a total of four samples. In the TAU group, two samples were obtained, aligning with the first and last time-points of MBGT + TAU.

**Table 3:** Sociodemographic variables at baseline for both conditions.

Variable	MBGT + TAU	TAU	X^2^/t (df)	p
	n / *mean* (SD)	n / *mean* (SD)		
Gender			0.38 (1)	.54
Male	13	14		
Female	12	9		
Non-binary	0	0		
Age	*45.16* (12.58)	*42.66* (12.45)	0.69 (46)	.49
Nationality			2.82 (2)	.25
German	20	22		
Turkish	1	0		
Other	4	1		
Family Status			1.06 (2)	.59
Single	17	18		
Married	3	1		
Divorced	5	4		
Widowed	0	0		
Living with partner	8	6	0.20 (1)	.65
With children	7	4	0.76 (1)	.38
Current housing situation			4.95 (3)	.18
Private flat	21	22		
Flat-sharing community	1	0		
Assisted living	3	0		
Other	0	1		
Years in school	*12.00* (1.32)	*11.75* (1.33)	0.65 (45)	.52
Highest educational achievement			5.48 (7)	.60
Primary school	1	0		
Lower secondary school	0	0		
Higher secondary school	5	5		
A level	3	5		
University degree	11	9		
Vocational training	3	3		
Without school-leaving qualification	2	0		
Other	0	1		
Occupation			1.19 (5)	.95
Unemployed	4	3		
In retirement	8	9		
Voluntary service	0	0		
Student	2	3		
Self-employed	3	2		
Employed	4	4		
Others	4	2		
Recruitment			0.76 (1)	.38
Intern	18	19		
Extern	7	4		
Diagnosis			1.85 (2)	.40
F20	16	15		
F23	2	0		
F25	7	7		

*Note.* P-values are based on Chi-square tests for categorial and *t*-tests for continuous variables; MBGT: mindfulness-based group therapy; TAU: treatment-as-usual; SD: standard deviation.

A rater assessed the outcome parameters using the Positive and Negative Syndrome Scale (PANSS) (Kay, Fiszbein & Opler, 1987) and the Personal and Social Performance Scale (PSP) (Nasrallah, Morosini & Gagnon, 2008) to determine positive- and negative symptoms and social functioning. These validated and highly reliable assessment tools are frequently employed in clinical trials and allow for a solid integration of the study results into the scientific context, as well as a detailed description of the clinical sample regarding symptom severity and social functioning.

Moreover, self-reported questionnaires were administered to assess empathy (Interpersonal Reactivity Index; IRI (Davis, 1980) & Empathy Quotient; EQ (Lawrence, Shaw, Baker, Baron-Cohen & David, 2004)), mindfulness (Southampton Mindfulness Questionnaire; SMQ (Böge et al., 2020b), and negative symptoms (Self-Evaluation of Negative Symptoms; SNS) (Dollfus, Mach & Morello, 2016). This study used the German short version of the Interpersonal Reactivity Index (IRI) (Paulus, 2009). While the SMQ has been specifically designed to assess mindfulness in individuals with psychosis (Böge et al., 2020b), the EQ and IRI assess participants' empathy levels. Moreover, the SNS provided further information on subjectively experienced negative symptom severity. The number of adverse events (AE) and serious adverse events (SAE) were assessed post-intervention. A detailed description of the assessments can be found in the appendix.

### Data management

All data collection and management were conducted using an electronic case report file (eCRF) based on the study software REDCap (Harris et al., 2019), located at the Charité – Universitätsmedizin Berlin. The electronic data collection has been set up and established and all study personnel involved in the data assessment and management received structured training. The eCRF software entails an authentication procedure, individual role management, and safe and encoded connections.

### Statistical analyses

Results were summarized by means/medians, standard deviations, and ranges for all baseline measures. Effect sizes were estimated for within- and between-group effects with corresponding confidence intervals. For clinical between-group differences, an ANCOVA was conducted with corresponding baseline scores as covariates. Within-group differences were analyzed using paired *t*-tests. For the analysis of OXT, within-group changes for MBGT + TAU were conducted by comparing pre-session and post-session levels at sessions one and four. In contrast, between-group analysis was conducted by comparing levels before sessions one and four, respectively. Effect sizes were reported using Cohen's *d* for within-group *t*-test and partial eta-squared for between-group ANCOVA. P-values were reported for two-sided testing. The groups were compared in terms of sociodemographic variables and medication regime using *t*-tests for continuous and chi-square tests for categorical variables. Due to the explanatory approach of the pilot study and the limited power, no corrections for multiple testing were conducted.

In general, as one goal of a pilot study is to gather preliminary data of outcome measures, which can be used to conduct a sample size calculation for a larger follow-up trial, the recommendation of approximately 30 participants per trial arm has been followed (Jacobsen et al., 2016; Jacobsen, Hodkinson, Peters & Chadwick, 2018; Lancaster, Dodd & Williamson, 2004). However, due to the COVID19-pandemic and related organizational burdens, this number could not have been reached in the pre-determined time frame. The significance level for alpha was set at 0.05. Statistical analyses were conducted using R Studio Version 2022.12.0 + 353 and IBM SPSS Statistics 27.

## Results

For the current study, 48 participants were recruited. The total sample consisted of *N* = 48 (21 women and 27 men), of whom 25 were randomized to MBGT + TAU and *n* = 23 to TAU. In comparison, 11 participants were recruited from external outpatient facilities, psychiatric and psychotherapeutic practices, assisted living facilities and psychiatric day hospitals. In terms of socio-demographic variables, the gender ratio was balanced across both groups: 13 men and 12 women in the MBGT + TAU condition and 14 men and nine women in the TAU condition. The mean age in MBGT + TAU was 45.16 years (*SD =* 12.58) compared to 42.66 years (*SD =* 12.45) in TAU. Overall, there were no significant differences in demographic and clinical characteristics at baseline between the two conditions. Table 3 shows a detailed description of the socio-demographic variables at baseline. In both groups, a similar share indicated to have prior experience in mindfulness practice. Current clinical care and treatment motivation at baseline are shown in Table A1.

Concerning the medication regime, there were statistically significant differences between the two conditions at baseline (*t*(1) = 6.01, *p* < .05) and post-intervention (*t*(1) = 7.24, *p* < .05) regarding the number of prescribed mood stabilizers. Specifically, more participants in the MBGT + TAU condition received mood stabilizers than participants in the TAU condition at both time points. The detailed medication regimen is described in Table A2. All means, and standard deviations for all measures at baseline and post-intervention in both conditions are shown in Table 4.

**Table 4:** Means and standard deviations of all measures for baseline and post-intervention in both groups.

Scales and subscales	T_0_		T_1_	
	MBGT+TAU	TAU	MBGT+TAU	TAU
	Mean (SD)	Mean (SD)	Mean (SD)	Mean (SD)
SMQ	47.41 (16.88)	49.00 (14.37)	52.68 (16.49)	49.95 (15.12)
*- Mindful observation*	12.91 (5.68)	13.00 (4.38)	14.95 (4.85)	13.04 (4.70)
*- Letting go*	10.27 (5.02)	11.36 (3.90)	12.09 (4.87)	11.38 (4.44)
*- Non-judgment*	11.32 (4.61)	11.09 (3.96)	12.23 (4.65)	12.14 (4.45)
*- Non-aversion*	12.91 (5.75)	13.55 (4.43)	13.41 (5.59)	13.59 (3.67)
CFQ	24.00 (10.70)	23.37 (8.15)	22.95 (8.54)	23.14 (8.67)
DASS – *depression*	6.05 (5.26)	5.52 (4.40)	6.00 (5.63)	4.87 (3.52)
DASS – *anxiety*	4.91 (4.47)	3.17 (2.89)	4.05 (3.47)	3.30 (2.42)
DASS – *stress*	6.05 (4.90)	6.00 (4.05)	5.95 (4.34)	5.52 (3.74)
EQ	39.14 (8.59)	36.57 (9.05)	41.09 (9.71)	37.04 (10.95)
SPF-IRI	41.09 (8.39)	40.05 (6.57)	39.68 (6.47)	40.90 (6.57)
- *Perspective Taking*	15.00 (3.21)	13.76 (2.53)	13.63 (2.89)	13.62 (2.09)
- *Fantasy*	11.59 (4.24)	12.14 (3.18)	11.50 (3.68)	12.70 (2.93)
- *Empathic Concern*	14.50 (2.86)	14.14 (2.31)	14.55 (2.24)	14.62 (1.83)
- *Personal Distress*	11.14 (3.20)	10.86 (2.99)	11.64 (2.24)	10.76 (2.68)
PSP	52.82 (12.75)	61.74 (12.48)	53.55 (13.68)	60.65 (11.74)
SNS	15.86 (7.62)	11.78 (6.97)	14.77 (8.55)	11.96 (6.19)
*- Social Withdrawal*	3.10 (2.28)	1.70 (1.74)	2.43 (1.96)	1.78 (1.57)
*- Diminished emotional range*	2.48 (2.18)	2.26 (1.45)	2.81 (2.40)	2.26 (1.89)
*- Alogia*	3.57 (2.38)	2.74 (2.47)	3.48 (2.48)	2.83 (2.19)
*- Avolition*	4.19 (2.34)	3.22 (2.47)	3.19 (2.42)	3.00 (1.95)
*- Anhedonia*	2.24 (2.17)	1.87 (1.60)	1.95 (1.75)	2.09 (1.44)
PANSS – *positive scale*	16.41 (5.97)	15.04 (5.51)	14.32 (5.02)	15.22 (6.24)
PANSS – *negative scale*	20.32 (4.91)	19.30 (5.78)	19.18 (6.42)	19.61 (6.77)

*Note.* MBGT: mindfulness-based group therapy; TAU: treatment-as-usual; SD: standard deviation; SMQ: Southampton Mindfulness Questionnaire; CFQ: Cognitive Fusion Questionnaire; DASS: Depression, Anxiety, Stress Scale; EQ: Empathy Quotient; SPF-IRI: Saarbrücker Persönlichkeitsfragebogen; PSP: Personal and Social Performance Scale; SNS: Self-Evaluation of Negative Symptoms; PANSS: Positive and Negative Syndrome Scale.

**Fig. 2:**
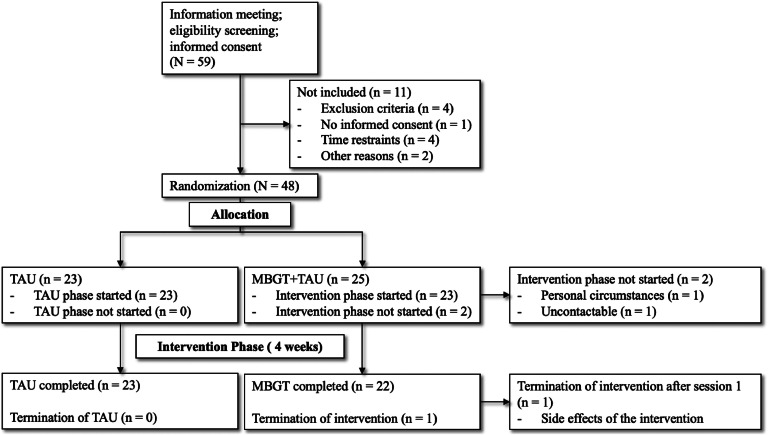
CONSORT flow diagram of the recruitment process.

The results show a 95.7% protocol adherence and a 94% retention rate regarding feasibility and acceptability. Notably, three individuals assigned to MBGT + TAU did not complete the post-intervention assessment, resulting in a 6% dropout rate. Furthermore, participants completed 95.65% of all sessions. One adverse event was reported in one patient who experienced an exacerbation of symptoms during the first session. Fig. 2 presents an overview of the CONSORT flowchart.

For the preliminary analysis of clinical outcomes, participants who completed the baseline and post-intervention assessment have been included (MBGT+TAU: *n* = 22; TAU: *n* = 23). Regarding empathy, within-subjects *t*-tests revealed no significant changes in MBGT + TAU between T_0_ and T_1_ for EQ, showing an increase of 1.95 points corresponding to a Cohen's *d* of 0.23. No significant difference could be found for the IRI or its subscales, with a mean decrease of 1.41 points in total score, corresponding with a Cohen's *d* of −0.25. Furthermore, no significant between-group difference at T_1_ regarding EQ and IRI and its subscales could be reported. Table 5 shows a detailed ANCOVA and post hoc *t*-tests overview.

**Table 5:** Within- and between group differences at the end for the primary outcome of empathy and the secondary outcomes.

Scales	Between group changes at T_1_			T_0_ to T_1_ for MBGT + TAU					T_0_ to T_1_ for TAU				
	F (*df*)	p	η_p_^2^	95% CI	t	p	Δ	d	95% CI	t	p	Δ	d
SMQ	1.21(1)	0.28	0.02	−2.05; 12.59	1.5	0.15	5.27	0.32	−1.62; 3.53	0.77	0.45	0.95	0.16
*- Mindful Observation*	2.32(1)	0.14	0.05	−0.85; 4.94	1.47	0.16	2.05	0.31	−1.22; 1.31	0.08	0.94	0.05	0.02
*- Letting Go*	1.93(1)	0.17	0.05	−0.54; 4.18	1.6	0.12	1.82	0.34	−1.17; 0.81	−0.38	0.71	−0.18	−0.08
*- Non-Judgment*	0.00(1)	0.95	0	−0.78; 2.60	1.12	0.28	0.91	0.24	−0.46; 2.55	1.45	0.16	1.05	0.31
*- Non-Aversion*	0.07(1)	0.79	0	−1.51; 2.51	0.52	0.61	0.5	0.11	−0.89; 0.98	0.1	0.92	0.04	0.02
CFQ	0.00(1)	0.96	0	−6.25; 4.15	−0.42	0.68	−1.05	−0.09	−2.82; 2.37	−0.18	0.86	−0.23	−0.04
DASS – *depression*	0.52(1)	0.48	0.01	−3.08; 2.99	−0.03	0.98	−0.05	−0.01	−2.02; 0.71	−0.99	0.33	−0.65	−0.21
DASS – *anxiety*	0.01(1)	0.93	0	−2.61; 0.88	−1.03	0.32	−0.86	−0.22	−1.16; 1.42	0.21	0.84	0.13	0.04
DASS – *stress*	0.14(1)	0.72	0	−2.92; 2.74	−0.07	0.95	−0.09	−0.01	−1.61; 0.65	.0.88	0.39	−0.48	−0.18
EQ	0.73(1)	0.4	0.02	−1.79; 5.70	1.09	0.29	1.95	0.23	−1.55; 2.51	0.49	0.63	0.48	0.1
SPF-IRI	2.47(1)	0.12	0.06	−3.95; 1.13	−1.15	0.26	−1.41	−0.25	−0.95; 2.67	0.99	0.34	0.86	0.22
- *Perspective Taking*	0.67(1)	0.42	0.02	−2.77; 0.05	−2	0.06	−1.36	−0.43	−1.02; 0.74	−0.33	0.74	−0.14	−0.07
- *Fantasy*	1.70(1)	0.2	0.04	−1.23; 1.04	−0.17	0.87	−0.09	−0.04	−0.39; 1.44	1.19	0.25	0.52	0.26
- *Empathic Concern*	0.31(1)	0.58	0.01	- 0.90; 0.99	0.1	0.92	0.05	0.02	−0.33; 1.29	1.27	0.23	0.48	0.27
- *Personal Distress*	1.76(1)	0.19	0.04	−0.68; 1.68	0.88	0.39	0.5	0.18	−0.83; 0.64	−0.27	0.79	−0.43	−0.06
PSP	0.03(1)	0.87	0	−4.83; 6.28	0.27	0.39	0.73	0.06	−4.41; 2.23	−0.68	0.5	−1.09	−0.14
SNS	0.28(1)	0.6	0	−4.07; 1.89	−0.76	0.46	−1.09	−0.16	−1.43; 1.77	0.23	0.82	0.17	0.05
*- Social Withdrawal*	0.05(1)	0.81	0	−1.63; 0.29	−1.45	0.16	−0.67	−0.32	−0.42; 0.59	0.36	0.72	0.09	0.08
*- Diminished Emotional Range*	0.78(1)	0.38	0.02	−0.50; 1.16	0.84	0.41	0.33	0.18	−0.57; 0.57	0	1	0	0
*- Alogia*	0.12(1)	0.73	0	−0.95; 0.76	−0.23	0.82	−0.1	−0.05	−0.40; 0.57	0.37	0.71	0.09	0.08
*- Avolition*	0.16(1)	0.69	0	−2.02; 0.02	−2.05	0.05	−1	−0.45	−0.74; 0.30	−0.87	0.4	−0.22	−0.18
*- Anhedonia*	0.36(1)	0.55	0.01	−0.98; 0.41	−0.86	0.4	−0.29	−0.19	−0.32; 0.75	0.84	0.41	0.22	0.18
PANSS – *Positive Scale*	6.27(1)	0.02	0.13	−3.65; −0.53	−2.79	0.01	−2.09	−0.59	−0.69; 1.04	0.42	0.68	0.17	0.09
PANSS – *Negative Scale*	0.62(1)	0.44	0.01	−3.59; 1.31	−0.97	0.35	−1.14	−0.21	−1.84; 2.45	0.29	0.77	0.31	0.06

*Note.* SMQ: Southampton Mindfulness Questionnaire; CFQ: Cognitive Fusion Questionnaire; DASS: Depression, Anxiety, Stress Scale; EQ: Empathy Quotient; SPF-IRI: Saarbrücker Persönlichkeitsfragebogen; PSP: Personal and Social Performance Scale; SNS: Self-Evaluation of Negative Symptoms; PANSS: Positive and Negative Syndrome Scale; PS: Positive Scale; NS: Negative Scale; CI: 95% Confidence Interval of the difference T_0_ – T_1_, η_p_^2^: partial eta-squared; Δ: mean difference score (T_1_ – T_0_); d: Cohen's d; Within-group test: paired-sample *t*-test; Between-group test: ANCOVA with respective baseline score as covariate.

Analysis of the OXT levels indicated that in blood plasma, levels increased within the first MBGT session increased (*M*_pre_ = 1.96pg/ml, *SD*_pre_ = 0.28pg/ml; *M*_post_ = 2.05, *SD*_post_ = 0.43, *d* *=* 0.4, *p =* .26), *M*_pre_ = 1.94, *SD*_pre_ = 0.19; *M*_post_ = 1.84, *SD*_post_ = 0.25, *d* *=* 0.18, *p <* .05). At the between-group level, both groups did not differ regarding baseline (T0) OXT levels. At T1, however, OXT levels in blood serum were significantly higher in TAU compared to MBGT+TAU (TAU: Mpost = 2.02, SDpost = 0.3), F(1,40) = 4.15, *p* < .05, η2 = 0.1).

Further findings regarding secondary outcomes showed that the SMQ measuring mindfulness did not change significantly within MBGT + TAU, as it increased by 5.27 points (*d* = 0.32). No significant changes could be reported within TAU, as the SMQ score increased by 0.95 points (*d* = 0.16). Regarding positive symptoms, participants in MBGT + TAU significantly decreased in PANSS positive scores by 2.09 points, corresponding to a Cohen's d of −0.59). In contrast, the TAU condition displayed a slight worsening of symptoms by 0.17 points (*d* = 0.09). The results for negative symptoms revealed that participants in MBGT + TAU showed statistically insignificant improvements from T0 to T1 in negative symptoms measured with the PANSS by 1.14 points (*d* = −0.21) and the self-assessment instrument SNS by −1.09 points (*d* = −0.16) compared to the TAU group that displayed an increase of negative symptoms by 0.17 on the PANSS (*d* = 0.06) and 0.17 points on the SNS (*d* = 0.05). Social functioning results revealed no significant within-subject effects for participants in the MBGT + TAU condition from T0 to T1 on the PSP, however, displaying a slight increase in social functioning by 0.73 points for MBGT+TAU (*d* = 0.06), while the mean score decreased in the TAU group by 1.09 (*d* = −0.14).

## Discussion

The objective of the current study was to assess the influence of MBGT on OXT levels and alterations in clinical parameters, both within and between groups as well as the further examination of feasibility and acceptability of the intervention in an outpatient setting. Compared to TAU alone, this evaluation was conducted at baseline and following a four-week MBGT intervention in conjunction with outpatient TAU.

At baseline, there were no statistically significant differences in demographic or clinical measurements between the two groups. Participants in MBGT + TAU received more mood stabilizers than participants in TAU at both time points. While higher p-values indicate biased randomization, ongoing debate challenges this heuristic (Stang & Baethge, 2018). Notably, MBGT + TAU participants appear to have slightly increased symptom severity. With a mean disorder duration of 16.22 years, MBGT + TAU represents a sample of outpatients with severely chronic disease courses. In addition, it has a total score of 15.57 points on the SNS and 20.32 points on the negative scale of the PANSS, indicating a severe manifestation of negative symptoms in MBGT + TAU at T_0_. In comparison, negative symptom severity was lower in TAU, with a total score of 11.78 points on the SNS and 19.30 points on the PANSS negative scale.

Outcomes of the current study suggest that implementing MBGT in the outpatient setting was a feasible, acceptable, and safe intervention, supporting the growing body of evidence that MBIs are appropriate and successfully implementable interventions in the management of SSD (Böge, Thomas and Jacobsen, 2021, Reich, Evans, Nelson, Hickey, & O’Shea, 2021). With a dropout rate of 6%, the results indicate a high level of participant engagement and satisfaction with the intervention. The results confirm high protocol adherence and retention among study participants. As the study progressed, adverse events and the side effect profiles of the intervention were assessed after each group session to ensure safety. One adverse event was reported in one patient who experienced an exacerbation of symptoms during the first session. However, this patient's high initial symptoms should be considered. Notably, this participant fulfilled inclusion criteria as no item on the PANSS has been scored < 6; however, there were multiple items on the PANSS positive scale on which the participant scored just below the cut-off. For future trials, it might be considered to adapt the inclusion criteria in that the sum score of the PANSS is determined as an inclusion criterion to ensure the participants’ safety during the MBI. No other events were reported. The results confirm that MBGT represents an indicated, safe, and well-tolerated treatment approach for persons SSD in the outpatient setting, even in a severely chronic sample, and contradict the myth of harmful effects on persons with SSD (Böge et al., 2021). However, future studies should incorporate more robust and objective instruments that assess the relatedness of adverse events to the intervention.

Previous research reported increased empathy in healthy individuals after the introduction of MBIs (Bellosta-Batalla et al., 2020, Centeno, 2020, Hu et al., 2022, Tan, Lo, & Macrae, 2014). Results in the current study indicated no significant within-subject or between-group effects on empathy in either condition. When analyzing previous research reporting increased empathy in healthy individuals, all studies applied compassion-based MBIs (Addington, Addington, Maticka-Tyndale, & Joyce, 1992, Bellosta-Batalla et al., 2020, Centeno, 2020, Hu et al., 2022, Tan, Lo, & Macrae, 2014). Because these interventions improve participants' theory of mind, their implementation can lead to a shift in empathy (Bellosta-Batalla et al., 2020, Mavituna et al., 2023). MBGT is based on a traditional understanding of mindfulness and focuses on mindfulness in the context of breathing, senses, distance, and body awareness. It was not specifically designed to improve empathy (Böge & Hahn, 2021). In addition, the therapeutic dose, including the duration of the therapy program, is to be discussed. Ridderinkhof, de Bruin, Brummelman, and Bögels (2017) examined the effects of short-term MBIs on empathy in healthy participants. The results showed no significant differences in empathy after participating in a one-time mindfulness exercise compared to participants who completed a control exercise (Ridderinkhof, de Bruin, Brummelman, & Bögels, 2017). Likewise, Centeno (2020) recommends a two-hour mindfulness practice twice weekly for eight weeks.

In addition to empathy, the current study examined changes in OXT levels at within- and between-group levels. During the first MBGT session, OXT levels increased, whereas during the fourth session, the levels decreased, which resulted in a significant between-group difference at T_1,_ with participants in MBGT+TAU displaying lower levels of OXT. These results align with previous research suggesting the role of OXT in social affiliation and stress regulation (Kubzansky, Mendes, Appleton, Block, & Adler, 2012, Linnen, Ellenbogen, Cardoso, & Joober, 2012). Participation in a group-therapy for the first time poses a new social and, therefore, stressful situation, which can explain the increase in OXT levels within the first session. In contrast, in the fourth session, the social stress of MBGT participants might have been lower, as fellow participants and the therapist were already more familiar, leading to lower OXT levels. This supports the idea of augmenting psychotherapeutic groups, such as MBGT, by administering exogenous oxytocin via nasal spray. Preliminary results of such an approach by our research group indicate an additional effect of OXT administration before MBGT, serving as a positive social context on psychopathology (Zierhut et al., 2024). However, consistent effect of intranasal oxytocin for the treatment of SSD could yet be shown by meta-analyses (Sabe, Zhao, Crippa, & Kaiser, 2021). Future studies should further enquire the association between OXT and stress in the context of MBGT and MBI in general.

Regarding the exploratory assessment of secondary outcomes, results differed from a feasibility trial in an inpatient setting (Böge et al., 2021). The sample in the current study displayed a long disorder duration and severe negative symptom severity, in which the aforementioned therapeutic dosage of four sessions might be insufficient to induce significant change. It is plausible that secondary negative symptoms were improved, while primary negative symptoms might not have shown significant changes. Reductions observed are likely more attributable to improvements in asociality and sedation. In the long run, the primary dimension of negative symptoms, such as motivation and diminished expression, may show reduction, but within a four-week timeframe, the observed effects might appear to be predominantly related to secondary negative symptoms. Another difference in outcomes compared to the feasibility trial is the non-significant increase in mindfulness as assessed by the SMQ. While the MBGT+TAU group had an increase of around five points on the SMQ, the TAU group displayed an increase of approximately one point between T0 and T1. In the feasibility trial, the scores were 43 and 45 for MBGT+TAU and TAU, respectively. The current sample displayed a higher baseline mindfulness-level with a score of 47 and 49 on the SMQ. This elevated baseline score might have led to a smaller increase in mindfulness skills based on four sessions of MBGT. A further likely explanation for these non-significant outcomes compared to the inpatient feasibility trial is the therapeutic dosage of the intervention. While the feasibility trial applied 12 sessions compared to the current study with only 33% (4/12) of the full MBGT program, other studies employing mindfulness-based interventions are set for 8 – 24 hours with an optimal dose of around 12 weeks (Sabe et al., 2024, Sabe, Sentissi, & Kaiser, 2019). These outcomes suggest that a higher therapeutic dose may be more appropriate, particularly in the outpatient setting, given the severe chronicity of the current sample. Nevertheless, consistent with the inpatient trial, the significant results and trends observed in the current study regarding mindfulness, social functioning, and both negative and positive symptoms indicate the beneficial effects of MBGT. These improvements regarding positive and negative symptoms are in line with previous meta-analytics research (Jansen et al., 2020). A higher sample size in further trials might confirm these trends and provide the required statistical power to determine the effects of these outcomes.

Furthermore, the sample of the current study, on average, improved 1.2 points on the negative syndrome scale of the PANSS. A change of 1.5 points has been found to correspond with a medium effect between minimally improved and unchanged patients (Czobor et al., 2022), which indicates that the current outcomes on negative symptoms are promising but once again highlight the importance of considering the treatment dosage according to the symptom severity. More specifically, regarding negative symptoms, the subscale Avolition of the SNS displayed the highest improvement during the intervention, underlining its role as a therapeutic mechanism already indicated by previous research group research (Zierhut et al., 2024).

Limitations of the trial comprise its design as a single-center trial, limiting the generalizability of the study’s outcomes and restricting the heterogeneity of the study’s sample. Statistically, a number of tests were employed due to the nature of this study being a pilot trial and to solidify the preliminary outcomes of this study, alpha adjustments in full trials should be employed to control the type-I error rate. Furthermore, a sample size of N = 48 only allows for a proof of concept and not to determine efficacy. As the nature of the current study is exploratory, no follow-up data has been examined, which further limits the outcomes. Also, dropouts have been excluded from the analyses of clinical parameters, which poses a risk of bias. Future studies should include an intention-to-treat approach to reduce this risk. Furthermore, the current sample was of relatively high age and displayed high negative symptoms, inhibiting the treatments' effect (Bighelli et al., 2023). Notably, a high proportion of participants in each group have a university degree, which might not be representative of the overall SSD population and result therefore need to be interpreted with caution. When pre-registered, a sample size of 60 was pursued, however due to the COVID19-pandemic and related organizational burdens, this target has not been reached. This might lead to inflated feasibility outcomes. Future trials should address these limitations while also considering the intervention's therapeutic dosage, which was arguably too small in the current study.

The strengths of the study design comprise the statistical analyses that assessed any deviations in received treatment between the active condition and TAU at baseline. Medication regimes were recorded at baseline and compared between groups. As no medication plan was adapted during the intervention phase, a confounding effect of this variable can be considered as unlikely. However, no differentiation has been made between first- and second- and third generation antipsychotics, and induced negative symptoms by high levels of D2 occupancy could not be ruled out (Sabe, Zhao, Crippa, & Kaiser, 2021). Additionally, medication in the number of mood stabilizers prescribed differed between both groups at both time points. Participants in the MBGT+TAU group were more likely to be prescribed a mood stabilizer. This difference might impact the results, particularly with regard to affective symptoms. Future studies with larger sample sizes should, therefore, include medication as a covariate in between-group analyses. Nevertheless, the monitoring restricts the heterogeneity of TAU and fosters replicability and generalizability of the study's outcomes while providing high-standard health care for all participants. In upcoming trials, the amount of received care between T0 and T1 should be recorded to monitor the received care outside the facility more thoroughly.

Overall, the results indicate the feasibility and acceptability of MBGT in an outpatient setting. Moreover, analyses indicate an effect of MBGT on oxytocin levels in individuals with SSD. While no effect was found on empathy levels, MBGT shows a moderate effect on positive symptoms which is in line with recent meta-analytics outcomes as well as improvements in negative symptoms, social functioning, and mindfulness on a within-group level. Although these signs are promising, it is important to note that further research is required, and fully powered RCTs are needed to consolidate the evidence.

## Author contributions

KB: conceptualization, funding acquisition, methodology, writing, supervision. NB: conceptualization, data curation, formal analysis, investigation, methodology, project administration, writing. MZ: conceptualization, methodology, resources, review and editing. IH: investigation, review and editing. AB: review and editing. JK: review and editing. IC: investigation, review and editing. TMTT: review and editing. NT: review and editing. PC: review and editing. SR: review and editing. EH: supervision, review and editing.

## Declaration of competing interest

The authors declare that they have no known competing financial interests or personal relationships that could have appeared to influence the work reported in this paper.

## Figures and Tables

**Table A1:** Clinical care and treatment motivation at baseline for both conditions.

	MBGT + TAU	TAU	χ^2^ / t (df)	p
	n / *mean* (SD)	n / *mean* (SD)		
Presence of psychotherapeutic treatment	9	6	0.55 (1)	.46
Individual therapy	9	6		
Group therapy	0	0		
Family therapy	0	0		
Couple therapy	0	0		
Frequency of therapeutic treatment			2.96 (2)	.23
Never	0	0		
One-time	2	1		
As required	3	0		
Weekly	4	5		
Daily	0	0		
Duration of therapeutic treatment in years	*6.22* (8.17)	*3.83* (5.01)	0.63 (12)	.54
Duration of illness in years	*16.22* (11.25)	*14.24* (10.41)	0.62 (44)	.54
Number of comorbid diagnoses			1.61 (3)	.66
0	20	21		
1	2	1		
2	2	1		
3	1	0		
Importance of therapy			5.56 (5)	.35
Very important	14	8		
Important	3	6		
Quite important	3	3		
Neutral	3	6		
Quite unimportant	1	0		
Unimportant	0	0		
Very unimportant	1	0		
Motivation			1.54 (5)	.91
Very motivated	9	7		
Motivated	7	6		
Quite motivated	6	5		
Neutral	2	3		
Quite unmotivated	0	1		
Unmotivated	1	1		
Very unmotivated	0	0		
Expectation of therapeutic success			2.80 (4)	.59
Very successful	7	6		
Successful	8	12		
Quite successful	6	3		
Neutral	3	1		
Quite unsuccessful	1	1		
Unsuccessful	0	0		
Very unsuccessful	0	0		
Previous experience with mindfulness	17	15		

*Note.* P-values are based on Chi-square tests for categorial and *t*-tests for continuous variables; MBGT: mindfulness-based group therapy; TAU: treatment-as-usual; SD: standard deviation.

**Table A2:** Medication regimen at baseline and post-intervention.

Type, number and dose equivalent of medication	Baseline				Post-intervention			
	MBGT+TAU	TAU	X^2^/t(df)	p	MBGT+TAU	TAU	X^2^/t(df)	p
	n / *mean* (SD)	n / *mean* (SD)			n / *mean* (SD)	n / *mean* (SD)		
Antipsychotics			1.45 (3)	.69			2.76 (4)	.60
0	2	1			3	2		
1	8	11			7	10		
2	13	10			11	10		
> 3	2	1			0	1		
Dose equivalent in mg^a^	*241.43* (216.78)	*256.63* (249.14)	2.26 (46)	.82				
Antidepressants			2.39 (3)	.50			0.47 (2)	.80
0	12	13			12	14		
1	12	9			8	8		
2	1	0			2	1		
> 3	0	1			0	0		
Mood stabilizers			6.01 (1)	**.01**			7.24 (1)	**.01**
0	17	22			16	23		
1	8	1			6	0		
Benzodiazepines			–	–			–	–
0	25	23			22	23		
1	0	0			0	0		

*Note.* Numbers refer to the n of participants in each group receiving the corresponding number of medications at the time points. P-values are based on Chi-square tests for categorical and *t*-tests for continuous variables; MBGT: mindfulness-based group therapy; TAU: treatment as usual; SD: standard deviation.

a Dosages were converted to Cariprazin.

## Appendix

### Assessment description

**Mindfulness.** We administered the self-reported Southampton Mindfulness Questionnaire (SMQ) (Böge et al., 2020b). This instrument measures an individual's level of mindfulness in response to distressing thoughts, images, or perceptions experienced over the past seven days. Mindfulness is conceptualized within the SMQ as comprising four interrelated aspects: 1) decentred awareness, 2) letting go, 3) non-judgment, and 4) non-aversion. The SMQ consists of 16 statements (e.g., 'I evaluate the thought/image as good or bad') that respondents rate using a seven-point Likert scale, ranging from 'completely agree' (6) to 'completely disagree' (0), with an additional option to select 'unsure' (3). It's worth noting that this 'unsure' option differs from the other two questionnaires used in this study, as it provides respondents with a neutral response choice (Chadwick et al., 2008). Consequently, the total SMQ score can vary between 0 and 96, with higher scores indicating a greater degree of mindfulness. Half of the questionnaire items are phrased positively, while the other half are phrased negatively. The German version of the SMQ exhibits good internal consistency, with a Cronbach's alpha value of 0.89 (Böge et al., 2020b).

**Cognitive Fusion.** Cognitive fusion was evaluated using the Cognitive Fusion Questionnaire (CFQ) (Gillanders et al., 2014), which comprises seven items, each rated on a 7-point Likert scale (1 = never true, 7 = always true). Greater scores indicate a stronger connection with inner experiences. The CFQ has demonstrated strong psychometric properties, as previous research shows, including high internal consistency (α = 0.90) (Gillanders et al., 2014).

**Depression, Anxiety, Stress.** To assess clinical outcomes and measure the severity of depression and anxiety symptoms, we employed the Depression, Anxiety, and Stress Scale (DASS-21). This questionnaire comprises 21 items, with seven items allocated to each of its three subscales: depression, anxiety, and stress. Notably, the anxiety subscale pertains to panic disorder, while the stress subscale is relevant to generalized anxiety disorder (Huppert & Smith, 2005). Respondents rated their responses on a four-point Likert scale, from (0) not applicable to me in the last week to (3) highly applicable to me in the last week. The DASS exhibits strong internal consistency with α values exceeding 0.80 across all three subscales. Multiple studies, including those by Page, Hooke and Morrison (2007) and Samson and Mallindine (2014) have demonstrated its effectiveness as a measurement tool for individuals with SSD (Page et al., 2007).

**Empathy.** The Empathy Quotient (EQ) is a self-report questionnaire comprising 60 items, with 40 dedicated to empathy assessment and 20 serving as control items (Baron-Cohen & Wheelwright, 2004). Respondents use a 4-point Likert scale in a forced-choice format, ranging from "strongly agree" to "strongly disagree." Individuals can score 2, 1, or 0 points for each empathy item, resulting in an EQ total score ranging from 0 to 80. To mitigate response bias, 19 of the 40 empathy items are phrased to elicit a "disagree" response for empathic behaviors. On these items, a response of "definitely disagree" awards 2 points, "slightly disagree" garners 1 point, and both "agree" responses yield 0 points. Conversely, on the remaining empathy items, "definitely agree" responses accrue 2 points, and "slightly agree" responses accumulate 1 point, while "definitely disagree" and "slightly disagree" responses each score 0 points. Baron-Cohen and Wheelwright (2004) underscored the EQ's straightforward scoring process, free from interpretation. They reported excellent test-retest reliability (*r* = 0.97), concurrent validity (Lawrence et al., 2004), as well as convergent and divergent validity. Internal consistency was high, with Cronbach's alpha at 0.92 (Baron-Cohen & Wheelwright, 2004).

The SPF-IRI (Paulus, 2009) represents the German adaptation of the Interpersonal Reactivity Index (Davis, 1980), a widely employed instrument for measuring empathy. This 16-item self-report questionnaire employs a five-point Likert format (1 = never, 5 = always). The items can be categorized into four subscales: Perspective Taking, Fantasy, Empathic Concern, and Personal Distress. The SPF-IRI generates individual scores for each subscale, as well as an overall empathy score. Paulus (2009) reported favorable reliability characteristics, satisfactory factorial validity, and item discrimination. Internal consistency was also robust, with Cronbach's alpha at 0.78 (Paulus, 2009).

**Social Functioning**. The Personal and Social Performance Scale (PSP) is a clinician-administered tool designed to gage the extent of personal and social dysfunction among individuals with SSD. It comprises four subscales, which assess socially useful activities, personal and social relationships, self-care, and disturbing and aggressive behaviors (Nasrallah et al., 2008). Each subscale is evaluated using a six-point rating format (1 = absent, 6 = very severe), culminating in an overall score ranging from 1 to 100. Higher scores indicate improved personal and social functioning. Nasrallah et al. (2008) reported favorable validity, inter-rater reliability, and test-retest reliability (ICC = 0.79) in patients with stable schizophrenia. Subsequent research revealed satisfactory internal consistency, with Cronbach's alpha ranging from 0.64 to 0.84 (Juckel et al., 2008).

**Positive and Negative Symptoms.** The Positive and Negative Syndrome Scale (PANSS) (Kay et al., 1987) involves a semi-structured psychiatric interview lasting 30 to 40 min. The scale encompasses 30 items, categorized into three subscales; in our study, we utilized the Negative Scale. This scale comprises seven statements that encompass blunted affect, emotional withdrawal, poor rapport, passive/apathetic social withdrawal, difficulty in abstract thinking, lack of spontaneity and flow of conversation, and stereotyped thinking. Interviewers assess each statement using a seven-point Likert format (1 = absent, 7 = extreme). Ratings are informed by clinical interviews and information provided by primary care staff or family members. The PANSS is a prominent instrument for assessing the prevalence of positive and negative syndromes in SSD. Kay et al. (1987) reported robust reliability and validity characteristics, including high interrater reliability and construct validity. For the Negative Scale, satisfactory test-retest reliability (*r* = 0.68) and internal consistency (α = 0.83) were demonstrated. Employing both self-evaluation and clinician-based measurements to assess negative symptom severity allows for a comprehensive understanding, incorporating both subjective experiences and objective clinical assessments of symptom severity.

Regarding negative symptoms, the Self-Report Negative Symptoms Scale (SNS) (Dollfus et al., 2016) is a self-assessment tool consisting of 20 items, employing a three-point Likert format (0 = strongly disagree, 2 = strongly agree). Respondents reflect on their feelings over the past week when evaluating each item. The cumulative score, derived from summing all items, ranges from 0 (indicating no negative symptoms) to 40 (reflecting severe negative symptoms). Four of these items contribute to each of the five subscales: Social Withdrawal, Diminished Emotional Range, Avolition, Anhedonia, and Alogia. Dollfus et al. (2016) reported robust psychometric properties, including high internal consistency (α = 0.87), strong convergent and discriminant validity, and substantial intrasubject reliability (ICC = 0.94).
